# Mindfulness in Technology: Feasibility and Preliminary Efficacy of VR‐Assisted Meditation Among Veterans With Disabilities

**DOI:** 10.1002/cpp.70266

**Published:** 2026-04-04

**Authors:** Uğur Doğan, Hung Jen Kuo

**Affiliations:** ^1^ Department of Guidance and Psychological Counseling Muğla Sıtkı Koçman University Muğla Turkiye; ^2^ Department of Counseling, Educational Psychology and Special Education Michigan State University East Lansing Michigan USA

**Keywords:** disabled veterans, meditation, mindfulness, psychological well‐being, technology usability, virtual reality

## Abstract

Veterans with physical disabilities may experience substantial psychological burden (e.g., PTSD symptoms) and practical barriers to accessing conventional mental health services. VR‐assisted meditation could offer a low‐threshold alternative, but evidence in this population remains limited. The objective of this study was to examine the feasibility, acceptability and preliminary efficacy of a VR‐assisted meditation programme for veterans with disabilities. In a randomized controlled trial, 40 US veterans (mean age = 42 years) were assigned to either (a) weekly VR‐assisted meditation sessions or (b) a no‐intervention control condition. Psychological well‐being (WHO‐5), mindfulness (SMS), system usability (SUS) and user satisfaction (QUEST 2.0) were measured at baseline, post‐intervention and follow‐up. Compared with controls, the VR group showed higher post‐intervention scores, with the clearest gains observed for psychological well‐being and perceived system usability. Scores decreased slightly at follow‐up but remained above the control condition. The pattern of benefits did not appear to differ by baseline anxiety level. VR‐assisted meditation may be a feasible and acceptable approach for supporting well‐being in veterans with disabilities. High perceived usability may support short‐term engagement; however, the present findings are preliminary and do not establish clinical effectiveness or durability.

## Summary

- VR‐assisted meditation showed short‐term benefits in psychological well‐being and user experience relative to a waitlist control, with attenuation at follow‐up.
- Mindfulness effects may not persist without repetition: Brief guided practice can improve state mindfulness, but sustained benefit likely requires ongoing, scheduled practice and integration into daily routines.
- Usability supports adherence: Strong gains in perceived usability (SUS) suggest that comfort, low set‐up burden and intuitive design can help participants return to practice over time.

## 1 Introduction

Veterans who acquire physical disabilities during military service often experience multiple, overlapping mental health difficulties, including post‐traumatic stress disorder (PTSD), depression, anxiety and social isolation (Hoge et al. 2004; Seal et al. 2009; Trivedi et al. 2015). These difficulties can reduce quality of life and limit social participation (Zinzow et al. 2012). They also raise societal costs through higher healthcare use and lost productivity (Greenberg et al. 2015). However, veterans may face obstacles to engaging with or staying in care. Concerns about stigma and other cultural factors can discourage help‐seeking, and logistical constraints (e.g., scheduling, transportation and time demands) may further limit engagement with evidence‐based psychological treatments (Kim et al. 2011).

Even when veterans are able to access care, outcomes are often modest. Standard pharmacological and psychosocial treatments show relatively low rates of full remission (Rush et al. 2006), and dropout remains common even in evidence‐based psychotherapies (Goetter et al. 2015; Hoge et al. 2014; Hundt et al. 2018). Complementary approaches such as meditation may be helpful for some individuals, but they can be difficult to deliver in ways that are accessible and sustainable for veterans with disabilities (Kabat‐Zinn 2013; Marchand 2013; Marchand et al. 2021). These factors motivate interest in formats that are easier to deliver and acceptable to users. In this study, we therefore focus on feasibility and short‐term outcomes rather than clinical effectiveness. This need is amplified by the heterogeneity of disabled veterans, who differ in age, type of disability (e.g., physical or sensory impairments) and military experiences. Effective programmes therefore need to be flexible and adaptable to individual circumstances rather than relying on a single, uniform format (Trivedi et al. 2015).

Against this backdrop, virtual reality (VR) has been proposed as a delivery format that may reduce some barriers to participation. VR‐based interventions may reduce session‐level burdens (e.g., travel time and environmental distractions) and support repeated practice in a controlled setting (Rizzo and Shilling 2017). VR may also reduce stigma‐related concerns by providing a more private and self‐directed setting, which can support initial engagement for engagement (Wilson et al. 2008). Another feature is the degree of control VR allows over the therapeutic environment. For individuals with trauma histories, predictable and adjustable settings may support a stronger sense of safety and help manage anxiety during practice. One commonly discussed feature of VR is immersion. By reducing competing external distractions, immersive environments may help direct attention toward the therapeutic task (Slater and Wilbur 1997). This may be especially relevant for veterans who struggle to sustain focus during conventional meditation or mindfulness exercises (Navarro‐Haro et al. 2017). In turn, greater attentional engagement may improve attitudes toward treatment and increase participation over time.

Combining VR with meditation may influence engagement and short‐term outcomes relative to non‐immersive delivery. One proposed explanation is that both modalities engage overlapping neurobiological systems involved in emotion regulation (Ladakis et al. 2024; Riva et al. 2019). Based on prior work, we outline a plausible pathway that can be tested in future mechanism‐focused trials. The high immersion provided by VR hardware may function as an environmental shield that reduces competing external distractions, which may be particularly helpful for veterans who report difficulty sustaining attention during mindfulness practice (Navarro‐Haro et al. 2017; Van Doren et al. 2024). This physical occlusion may facilitate a stronger sense of presence—the psychological sensation of ‘being there’ (Slater and Wilbur 1997). This pathway is conceptual and was not tested as a mediator in the present trial. We propose that heightened presence may reduce the cognitive effort required to maintain attentional stability and support more consistent present‐moment awareness (Tarrant et al. 2018). By easing the cognitive load associated with sustaining attention, VR may enable more direct engagement with emotion‐regulation processes during mindfulness practice, which we hypothesize as a plausible pathway to improvements in psychological well‐being (Ladakis et al. 2024). Meditation content that targets stress reduction and emotional modulation can then be delivered through a platform that may be more accessible and acceptable for some users (Goyal et al. 2014; Navarro‐Haro et al. 2017), with early supportive findings in workplace samples that included employees with disabilities (Kuo et al. 2025). Additional VR features may further strengthen specific meditation techniques. For example, VR‐based embodiment may amplify interoceptive cues, which could enhance body‐scan practices (Gibson 2024; Bosman et al. 2025). Likewise, immersive scenes may make guided imagery more engaging and easier to sustain. This type of structure may be particularly useful for veterans who are hesitant to participate in trauma‐focused interventions, offering a less confrontational entry point to skill building (Badola et al. 2025). Finally, integrating real‐time biofeedback within VR could support physiological self‐regulation by reinforcing parasympathetic activation during practice (Spiegel et al. 2019). Taken together, these elements motivate feasibility work on VR‐assisted meditation for veterans with disabilities.

However, the empirical literature on VR‐based psychological interventions remains more nuanced than these advantages alone might imply, with mixed and sometimes null findings across outcomes and contexts. Meta‐analytic evidence suggests that VR interventions tend to outperform passive or waitlist controls, yet they do not consistently demonstrate superior outcomes relative to established active treatments, nor do they reliably reduce attrition (Fodor et al. 2018). Moreover, synthesis work indicates that dropout may, in part, reflect VR‐specific tolerability barriers—most notably cybersickness and difficulties achieving sufficient immersion—underscoring that immersive delivery is not uniformly acceptable for all users (Benbow and Anderson 2019). Importantly, many trials in this area are constrained by small samples, variable risk of bias and incomplete reporting (including effect sizes for active comparisons), which limits inferences about the clinical magnitude and generalizability of VR effects (Fodor et al. 2018). Taken together, this complexity strengthens—not weakens—the rationale for the present study: Rigorous feasibility research is particularly necessary in vulnerable populations such as veterans with disabilities, where acceptability, tolerability and sustained engagement with immersive technology cannot be assumed.

To clarify the contribution of the present study, our aim is primarily exploratory and applied, addressing a specific methodological gap in the emerging VR–mindfulness literature: whether a brief, guided VR‐assisted meditation protocol is feasible and acceptable for veterans with disabilities, and whether it shows preliminary signals of benefit on psychological well‐being. Importantly, we conceptualize this intervention not as an acute psychiatric treatment for severe PTSD, but rather as a low‐threshold, preventive approach. By targeting a subclinical sample with mild anxiety or stress, we aim to establish foundational feasibility and tolerability parameters before testing the protocol in clinical samples with clinician‐verified diagnoses. Rather than offering a purely confirmatory test of established efficacy, we extend prior work by focusing on a population with distinctive functional needs and a low‐incidence group that is difficult to recruit for research, while simultaneously evaluating technology experience (usability and satisfaction) alongside psychological outcomes. In doing so, the study is intended to provide foundational evidence that can inform the design of subsequent fully powered trials with active comparators and longer follow‐up, thereby informing trial design and implementation choices.

Accordingly, we conducted a two‐arm randomized study to evaluate the feasibility and acceptability of a brief VR‐assisted guided meditation programme for veterans with disabilities, alongside preliminary effects on psychological well‐being. This focus responds to two persistent gaps in the broader literature: first, the limited and largely pilot‐level evidence for mindfulness‐based programmes tailored to veterans with disabilities (Gallegos et al. 2017; Badola et al. 2025); and second, the predominance of disorder‐specific VR protocols in military mental health, with comparatively fewer trials targeting broader well‐being outcomes and implementation‐relevant endpoints (e.g., usability and user experience) in a low‐incidence, hard‐to‐recruit clinical–community group (Bouchard et al. 2017; Carl et al. 2019; Morina et al. 2023; Botella et al. 2015). In addition to psychological outcomes, we tracked technology experience longitudinally using both general usability (SUS) and assistive‐technology satisfaction (QUEST 2.0), which is rarely reported together in VR–mindfulness trials. By jointly assessing well‐being outcomes and technology experience, the study aims to provide a clearer empirical foundation for whether VR‐assisted meditation can be considered a scalable, acceptable adjunct for rehabilitation‐oriented mental health support among disabled veterans.

### 1.1 Primary Research Question

Does VR‐assisted meditation lead to significant improvements in psychological well‐being and mindfulness among disabled veterans compared to a control group, when assessed across baseline (T0), post‐test (T1) and follow‐up (T2) time points?

### 1.2 Secondary Research Questions (Construct‐Based)

Does the VR‐assisted meditation intervention result in improvements in perceived system usability and technology satisfaction at post‐test (T1) and follow‐up (T2) compared to the control group?

Are any observed improvements in usability and satisfaction sustained from post‐test (T1) to the follow‐up period (T2)?

### 1.3 Moderating/Control Variable Questions

When controlling for anxiety and stress, does VR‐assisted meditation show similar time × group patterns for psychological well‐being, mindfulness, system usability and technology satisfaction across T0, T1 and T2?

## 2 Materials and Methods

### 2.1 Research Design

This two‐arm randomized controlled trial evaluated the feasibility, acceptability and short‐term outcomes of a VR‐assisted guided meditation programme for veterans with disabilities. The trial was conducted in Michigan, a Midwestern state in the United States, and coordinated by Michigan State University. Participants in the intervention arm received guided meditation sessions via a commercially available application (Guided Meditation VR, guidedmeditationvr.com) once per week for five consecutive weeks; each session lasted approximately 20–25 min and took place in participants' homes, local libraries or at the Michigan State University library. The control arm received no intervention during the study period and was offered access to the VR programme after completion of follow‐up (T2) assessments. Data were collected at three time points—baseline (T0, Week 0), post‐test (T1, Week 5) and follow‐up (T2, Week 8)—using validated self‐report instruments assessing psychological well‐being, perceived stress, anxiety, mindfulness, system usability and technology satisfaction. The study complied with the Declaration of Helsinki and received approval from the Michigan State University Institutional Review Board (IRB); written informed consent was obtained from all participants. The protocol was developed in accordance with the SPIRIT 2013 checklist (Chan et al. 2013), and reporting adhered to CONSORT 2010 guidelines.

#### 2.1.1 Randomization and Masking

Participants were recruited using purposive sampling with peer‐referral (snowball) procedures. Interested individuals completed an online screening form (Microsoft Forms). Eligible participants were randomly assigned (1:1) to either the intervention or the waitlist control group using a computer‐generated block randomization sequence (block size of 4). Allocation concealment was ensured via centralized, web‐based randomization on a secure server, and group assignments were released only after the completion of T0 measures. A waitlist (no intervention) control was used because the study was designed as an initial feasibility trial; active‐comparator testing is planned for subsequent, fully powered studies. Given the nature of the intervention, participants and interventionists were not blinded. Because outcomes were self‐administered via online questionnaires, there were no independent outcome assessors; however, data were exported with masked group labels, and primary analyses were conducted before group labels were revealed. Participant confidentiality and data security were maintained throughout (coded IDs; encrypted, access‐restricted storage). The trial adhered to a prespecified protocol; no interim analyses were conducted, and there were no early stopping procedures.

### 2.2 Participants

**FIGURE 1:**
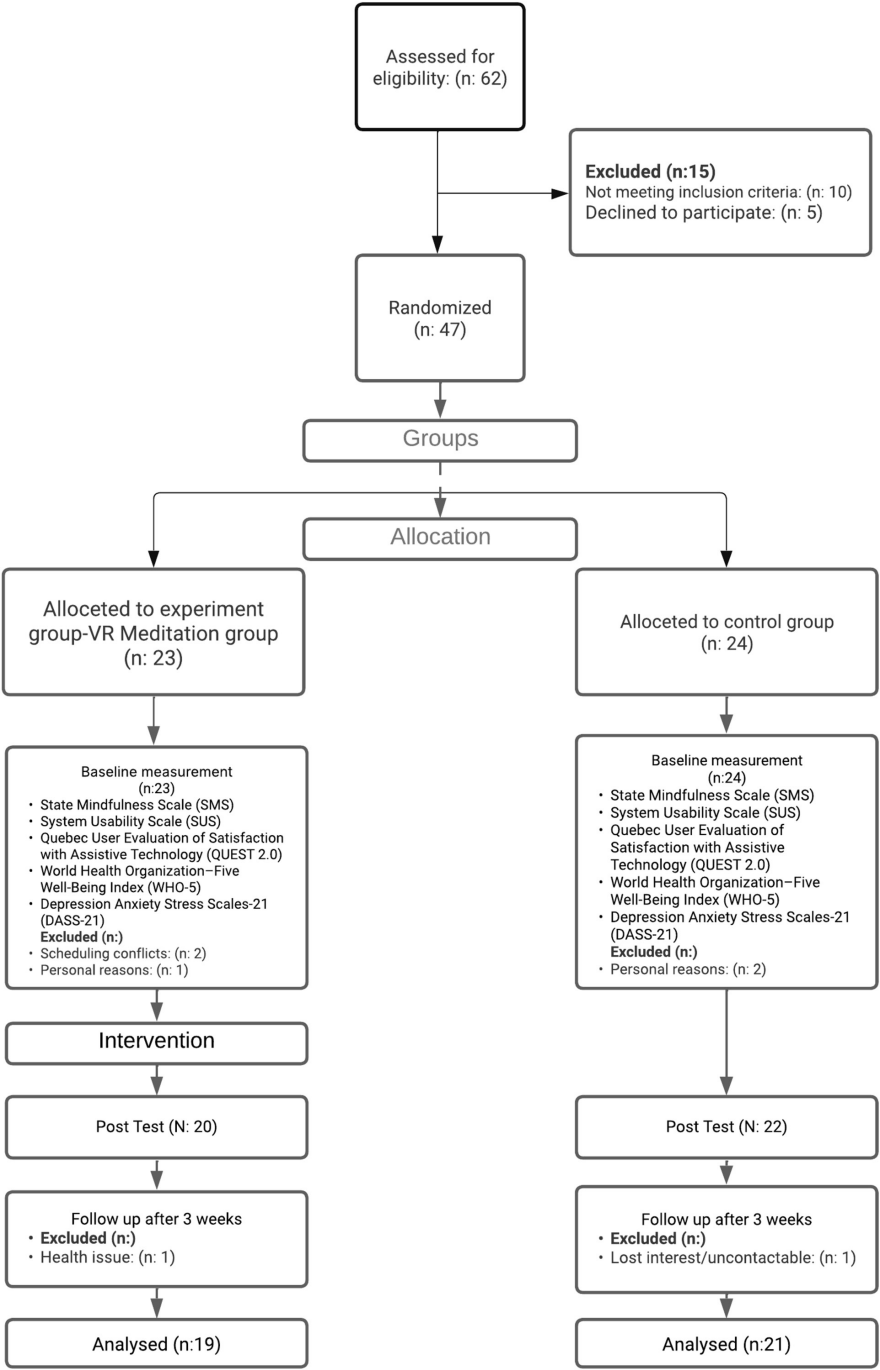
The flowchart of the experimental procedure.

A total of 62 individuals were screened; 47 met eligibility criteria and were randomized (23 intervention; 24 control). Seven participants were lost to follow‐up, yielding a final analysed sample of 40 (19 intervention; 21 control). The mean age was 42.10 years (SD = 4.04; range 35–50); 55.0% identified as male (*n* = 22) and 45.0% as female (*n* = 18). Inclusion criteria were as follows: disabled veteran status; no current mental‐health treatment; no clinician‐verified psychiatric diagnosis (e.g., PTSD, depressive or anxiety disorders); mild subclinical anxiety or stress (defined a priori using DASS‐21 severity bands); no prior experience with meditation; no prior exposure to VR applications; and provision of written informed consent. We also limited eligibility to subclinical levels to avoid creating unrealistic expectations of treatment benefit for individuals with severe, clinician‐diagnosed disorders (e.g., PTSD) in the context of an exploratory feasibility study. The decision to recruit a subclinical/preventive sample with mild anxiety rather than a clinical sample with severe, clinician‐verified PTSD was intentional. As an exploratory feasibility study, evaluating the intervention in a subclinical group mitigates the potential clinical risks associated with highly immersive environments (e.g., cybersickness or increased discomfort in highly immersive environments) and establishes foundational tolerability. Consequently, while these findings provide crucial preliminary evidence, they are primarily generalizable to early‐intervention or subclinical contexts rather than acute psychiatric treatment. Accordingly, we do not generalize these results to veterans with diagnosed PTSD or major depressive disorder; those populations require separate trials with clinician‐verified assessments and longer follow‐up. Participant flow is detailed in the CONSORT diagram (Figure 1).

### 2.3 Power Analysis

The required sample size was estimated with G*Power 3.1 (Faul et al. 2007) for a two‐group repeated‐measures ANCOVA with three measurement points (baseline, post‐test and follow‐up). Based on meta‐analytic findings on VR interventions for anxiety and depression (Fodor et al. 2018), we assumed a medium effect (Cohen's *f* = 0.25), α = 0.05 and power (1 − β) = 0.80. This indicated a minimum total sample of 28 participants (14 per group) to detect a group × time interaction. However, in view of previously reported high attrition rates in VR studies involving veteran populations—ranging from 15% to 39% (Beidel et al. 2019; McLay et al. 2011; Reger et al. 2011)—the study was initiated with a larger sample of 47 participants. This approach ensured that the final analysed sample size exceeded the predetermined minimum requirement. This allowed the analysed sample to exceed the minimum target, improving precision for the planned estimates.

### 2.4 Intervention Procedure

The VR‐assisted meditation intervention was delivered over 5 weeks, with one session per week. Sessions were scheduled according to participant availability and conducted in quiet settings (e.g., participants' homes or local libraries) to ensure comfort and privacy. Each session lasted approximately 20–25 min and followed a standardized structure: a single guided VR meditation practice of about 15–20 min, followed by a brief 3–5‐min debrief. Session structure and total duration were fixed across participants to support intervention fidelity. Before the first session, participants received a hands‐on orientation to the headset and application (Guided Meditation VR), including how to don and adjust the headset, launch and pause sessions and navigate within the software. To foster engagement while preserving fidelity, personalization was limited to non‐core elements (e.g., selecting from predefined virtual environments, guided prompts and musical tracks); the number of practices (one) and the session duration (20–25 min) were held constant. Participants were informed about potential adverse effects (e.g., cybersickness such as dizziness or nausea) and advised that they could pause or discontinue a session at any time if discomfort arose. Adverse events were recorded using a prespecified adverse‐event log. Safety procedures were followed throughout.

### 2.5 Equipment and Software

The intervention was delivered using the Meta Quest 2 standalone head‐mounted display (Reality Labs, Meta Platforms) and the Guided Meditation VR application (Cubicle Ninjas; accessed January 2025). The headset was used in standalone mode with six‐degrees‐of‐freedom inside‐out tracking; interpupillary distance (IPD) was adjusted individually. Guided Meditation VR provides nature‐based immersive scenes and guided prompts; to preserve intervention fidelity, session structure and duration (20–25 min) were fixed, while personalization was limited to non‐core elements (predefined environments/tracks). This set‐up allowed sessions to be delivered in home or community settings after a brief orientation.

### 2.6 Data Collection Process

Quantitative self‐report methods were used to assess the psychological impacts of the VR‐assisted intervention. Data were collected at three time points: baseline (T0, Week 0), post‐intervention (T1, Week 5) and follow‐up (T2, Week 8). Self‐report instruments were administered at each time point to assess psychological well‐being, perceived stress, anxiety, mindfulness, system usability and technology satisfaction. The primary outcomes were psychological well‐being (World Health Organization–Five Well‐Being Index, WHO‐5) and state mindfulness (State Mindfulness Scale, SMS). Technology‐related outcomes comprised perceived system usability (System Usability Scale, SUS) and technology satisfaction (Quebec User Evaluation of Satisfaction with Assistive Technology, QUEST 2.0). In addition, anxiety and perceived stress were assessed with the Depression Anxiety Stress Scales‐21 (DASS‐21). These variables were specified a priori as covariates in the primary analyses and examined as potential moderators in sensitivity analyses. Scoring and interpretation followed published guidelines for each instrument.

### 2.7 Measurements and Outcomes

Table 1 summarizes the measures and assessment schedule; all instruments were administered online at T0, T1 and T2. The primary outcomes were psychological well‐being (WHO‐5) and state mindfulness (SMS). Technology‐related outcomes comprised perceived system usability (SUS) and technology satisfaction (QUEST 2.0). Anxiety and perceived stress (DASS‐21 subscales) were specified a priori as covariates and were also examined as potential moderators in sensitivity analyses. Table 1 summarizes the instruments and the assessment schedule.

**TABLE 1:** Measurement tools and the points at which each assessment is made.

Construct	Instrument	T0	T1	T2
Mindfulness	SMS	X	X	X
System usability	SUS	X	X	X
Technology satisfaction	QUEST 2.0	X	X	X
Psychological well‐being	WHO‐5 Well‐Being Index	X	X	X
Anxiety (control variable)	Anxiety subscale of DASS	X	X	X
Stress (control variable)	Stress subscale of DASS	X	X	X

*Note:* T0 = baseline (Week 0); T1 = post‐test (end of Week 5); T2 = follow‐up (Week 8).

Abbreviations: DASS‐21, Depression Anxiety Stress Scales‐21; QUEST 2.0, Quebec User Evaluation of Satisfaction with Assistive Technology; SMS, State Mindfulness Scale; SUS, System Usability Scale; WHO‐5: World Health Organization–Five Well‐Being Index.

After scoring and data checks, data were prepared for the planned group × time analyses (repeated‐measures ANCOVA), adjusting for the prespecified covariates.

### 2.8 Measurement Instruments

#### 2.8.1 SMS (Tanay and Bernstein 2013)

The SMS assesses state mindfulness via two components: (1) attention to and awareness of present‐moment bodily and mental experience, and (2) a curious, accepting attitude toward that experience. It comprises 21 items (six bodily sensations; 15 mental events) rated on a 5‐point Likert scale (1 = *never*, 5 = *always*). Scores are typically computed as the sum (range 21–105) or the mean (range 1–5); higher scores indicate greater state mindfulness. In the current study, internal consistency (McDonald's ω) was 0.76 for the total scale, 0.73 for the mind subscale and 0.44 for the body subscale. (*In this study, we treated SMS as a continuous outcome using the conventional scoring; rescaling to 0–100 was not applied*).

#### 2.8.2 SUS (Brooke 1996)

The SUS is a 10‐item instrument rated on a 5‐point Likert scale (1 = *strongly disagree*, 5 = *strongly agree*) with alternating polarity items. After standard scoring, values are transformed to a 0–100 scale; ~68 is commonly cited as an average benchmark. Reported internal consistency is high (α ≈ 0.85–0.91). The internal consistency (McDonald's ω) for this baseline expectation‐based administration was satisfactory (ω = 0.81). (*We analysed SUS as a continuous outcome rather than using categorical cut‐offs. At T0, items were minimally adapted to capture pre‐use expectations; details and reliability are reported in the Results*).

#### 2.8.3 QUEST 2.0 (Demers et al. 1996)

QUEST 2.0 measures satisfaction with assistive technology across 12 items: eight device attributes (e.g., size, weight, adjustability, safety, durability, ease of use, comfort and effectiveness) and four service aspects (service delivery, repairs/maintenance, professionalism and follow‐up). Items are rated on a 5‐point scale (1 = *not at all satisfied*, 5 = *very satisfied*), and respondents identify their three most important items. The internal consistency (McDonald's ω) for the T0 expectation‐based administration in our sample was modest (total scale = 0.72; assistive device subscale = 0.69, services subscale = 0.70). (*At T0, we used a pre‐use expectation wording; post‐use versions were administered at T1 and T2*).

#### 2.8.4 WHO‐5 (WHO Regional Office for Europe, 1998)

The WHO‐5 assesses subjective well‐being over the past 2 weeks using five items rated on a 6‐point scale (0 = *at no time* to 5 = *all of the time*). The raw total (0–25) is multiplied by 4 to yield a 0–100 score; higher values indicate better well‐being. The internal consistency for the current sample was McDonald's ω = 0.51. Given the small sample, this estimate should be interpreted cautiously.

#### 2.8.5 DASS‐21 (Lovibond and Lovibond 1995)

The DASS‐21 comprises 21 items (seven per subscale: Depression, Anxiety, Stress) rated 0–3. Subscale totals are multiplied by 2 (range 0–42) for interpretation against established severity bands (normal, mild, moderate, severe and extremely severe). Consistency estimates are generally acceptable to high (e.g., α/ω ≈ 0.75–0.90, depending on subscale/sample). In this study, the Anxiety and Stress subscales were prespecified as covariates (and explored as moderators) in the primary analyses; a total DASS score was not used. The internal consistency in the current sample was McDonald's ω = 0.64 for the Stress subscale and ω = 0.40 for the Anxiety subscale.

### 2.9 Data Analysis

The primary analysis used a two‐arm, three‐time‐point repeated‐measures ANCOVA to test the group × time (T0, T1 and T2) interaction for each outcome. Baseline DASS‐21 Anxiety and Stress were specified a priori as between‐subject covariates. Model assumptions (normality of residuals, homogeneity of variances and homogeneity of regression slopes) were examined; where sphericity was violated, Greenhouse–Geisser corrections were applied. For significant interactions, estimated marginal means and pairwise contrasts (Tukey adjustment) were obtained across time points. Baseline comparability between groups was described using standardized mean differences (SMDs) rather than null‐hypothesis tests; no inferential testing of baseline characteristics was undertaken. All analyses were conducted in jamovi 2.5 (The jamovi project, 2024) and R (R Core Team, 2023). The afex package (Singmann et al. 2025) was used for repeated‐measures ANCOVA with Type‐III sums of squares and Greenhouse–Geisser corrections, and emmeans (Lenth 2023) was used to compute estimated marginal means and contrasts. The significance level was set at α = 0.05 (two‐tailed).

## 3 Results

Table 2 summarizes descriptive statistics for WHO‐5, QUEST 2.0, SMS and SUS by group at baseline (T0), post‐test (T1) and follow‐up (T2). These descriptives provide context for the group × time analyses reported in Table 3.

**TABLE 2:** Descriptive statistics of experimental and control groups at baseline measurement, post‐test and follow‐up.

Measurement	Group	Variable	M	Median	SD	Min	Max
Baseline measurement	Experimental group	WHO‐5	38.737	36	9.620	28	56
		QUEST	55.474	55	1.504	52	58
		SMS	38.842	35	8.783	29	55
		SUS	73.421	72.5	7.370	65	95
	Control group	WHO‐5	39.429	40	4.611	32	48
		QUEST	55.143	56	1.526	51	57
		SMS	41.333	41	1.826	39	45
		SUS	72.262	72.5	9.247	57	90
Post‐test	Experimental group	WHO‐5	44.421	44	6.095	36	56
		QUEST	58.158	58	1.302	56	60
		SMS	41.211	39	9.022	25	56
		SUS	86.053	85	2.924	80	90
	Control group	WHO‐5	39.048	40	4.364	32	48
		QUEST	55.333	56	1.528	52	58
		SMS	39.238	40	2.508	35	43
		SUS	72.238	75	10.733	57	92
Follow‐up	Experimental group	WHO‐5	41.895	40	7.817	32	60
		QUEST	57.053	57	1.545	54	59
		SMS	40.895	37	8.762	31	57
		SUS	83.921	90	8.335	70	92
	Control group	WHO‐5	39.429	40	4.057	32	48
		QUEST	54.714	55	1.488	52	57
		SMS	41.429	41	1.805	39	45
		SUS	70.857	70	9.774	57	87

Abbreviations: M, mean; Max, maximum; Min, minimum; QUEST, Quebec User Evaluation of Satisfaction with Assistive Technology 2.0; SD, standard deviation; SMS, State Mindfulness Scale; SUS, System Usability Scale; WHO‐5, World Health Organization–Five Well‐Being Index.

**TABLE 3:** Repeated‐measures ANCOVA results for WHO‐5, QUEST, SMS and SUS.

Measure	Factor	SS	df	MS	*F*	*p*	ƞp^2^
WHO‐5	Time	124.537	2	62.268	4.933	0.010	0.12
	Time × Group	215.115	2	107.557	8.521	< 0.001	0.19
	Time × Stress	67.092	2	33.546	2.658	0.077	0.07
	Time × Anxiety	16.631	2	8.315	0.659	0.521	0.02
	Residual	908.823	72	12.623	—	—	—
QUEST	Time	2.490	2	1.245	1.667	0.196	0.04
	Time × Group	35.524	2	17.762	23.778	< 0.001	0.40
	Time × Stress	0.919	2	0.460	0.615	0.543	0.02
	Time × Anxiety	1.494	2	0.747	1.000	0.373	0.03
	Residual	53.783	72	747	—	—	—
SMS	Time	1.176	2	0.588	0.183	0.833	0.01
	Time × Group	49.266	2	24.633	7.664	< 0.001	0.18
	Time × Stress	11.162	2	5.581	1.737	0.183	0.05
	Time × Anxiety	10.443	2	5.221	1.625	0.204	0.04
	Residual	231.405	72	3.214	—	—	—
SUS	Time	201.551	2	100.776	4.437	0.015	0.11
	Time × Group	1078.751	2	539.376	23.750	< 0.001	0.40
	Time × Stress	20.674	2	10.337	0.455	0.636	0.01
	Time × Anxiety	67.271	2	33.635	1.481	0.234	0.04
	Residual	1635.165	72	22.711	—	—	—

Abbreviations: df, degrees of freedom; MS, mean square; ƞp2, partial eta square; QUEST, Quebec User Evaluation of Satisfaction with Assistive Technology 2.0; SMS, State Mindfulness Scale; SS, sum of squares; SUS, System Usability Scale; WHO‐5, World Health Organization–Five Well‐Being Index.

Table 2 summarizes descriptive statistics (M, Median, SD, Min and Max) for WHO‐5, QUEST 2.0, SMS and SUS by group across baseline, post‐test and follow‐up. At baseline, group means were similar for WHO‐5, QUEST 2.0 and SUS; the control group had a higher SMS mean (M = 41.333) than the experimental group (M = 38.842). From baseline to post‐test, the experimental group showed increases on WHO‐5 (M = 44.421), QUEST 2.0 (M = 58.158) and SUS (M = 86.053), whereas the control group remained close to baseline. At follow‐up, experimental‐group means declined slightly relative to post‐test but remained higher than the control group; the control group was comparatively stable across phases, with notably lower SUS (M = 70.857). Differences on QUEST 2.0 and SMS were smaller in magnitude than those observed for WHO‐5 and SUS. To evaluate statistical significance of group‐by‐time differences, a repeated‐measures ANCOVA was conducted; results are reported in Table 3.

**FIGURE 2:**
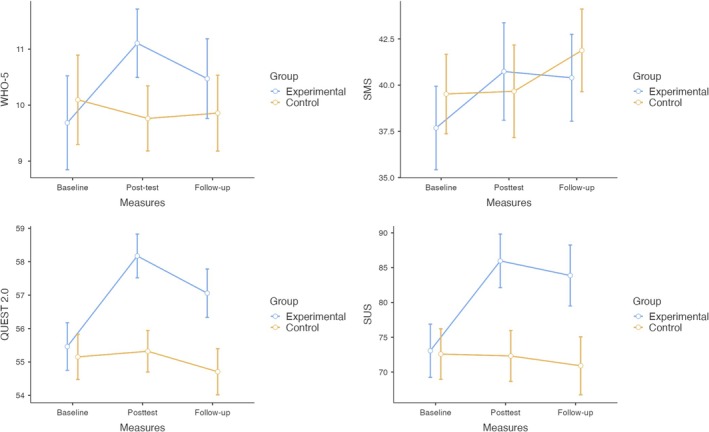
Adjusted means from the repeated‐measures ANCOVA.

Table 3 presents the results of the repeated‐measures ANCOVA conducted on WHO‐5, QUEST, SMS and SUS scores. For the primary time × group interactions, effect sizes were ηp^2^ = 0.19 (WHO‐5), 0.40 (QUEST), 0.18 (SMS) and 0.40 (SUS). For the WHO‐5 measure, the main effect of time was statistically significant (*F*_(2, 72)_ = 4.933, *p* = 0.010), indicating that well‐being changed over time. Additionally, the time × group interaction was also significant (*F*_(2, 72)_ = 8.521, *p* < 0.001), suggesting that the pattern of change in WHO‐5 scores differed between the experimental and control groups. However, the interactions of time × stress (*p* = 0.077) and time × anxiety (*p* = 0.521) were not significant, indicating no evidence that change over time differed as a function of stress or anxiety. In the QUEST measure, while the main effect of time was not significant (*F*_(2, 72)_ = 1.667, *p* = 0.196), the time × group interaction was statistically significant (*F*_(2, 72)_ = 23.778, *p* < 0.001), indicating that satisfaction with assistive technology increased more markedly in the experimental group compared to the control group over time. The interactions with stress (*p* = 0.543) and anxiety (*p* = 0.373) were not significant. For the SMS measure, the main effect of time was not significant (*F*_(2, 72)_ = 0.183, *p* = 0.833), whereas the time × group interaction was significant (*F*_(2, 72)_ = 7.664, *p* < 0.001), showing that changes in mindfulness differed between the experimental and control groups. Neither stress (*p* = 0.183) nor anxiety (*p* = 0.204) significantly interacted with time, indicating that no evidence that change over time differed as a function of stress or anxiety. In terms of SUS, the main effect of time was significant (*F*_(2, 72)_ = 4.437, *p* = 0.015), indicating a change in system usability perceptions over time. The time × group interaction was also significant (*F*_(2, 72)_ = 23.750, *p* < 0.001), indicating improved perceived usability among participants in the experimental group. Stress (*p* = 0.636) and anxiety (*p* = 0.234) interactions were not significant. Model assumptions were checked; where sphericity was violated, Greenhouse–Geisser corrections were applied. To decompose the significant time × group interactions identified in the main analysis, post hoc pairwise comparisons with Tukey's adjustment were conducted. For psychological well‐being (WHO‐5), the experimental group showed a significant improvement from baseline to post‐test (*p* < 0.001) and scored significantly higher than the control group at post‐test (*p* = 0.029) (see Table S1). For SUS and QUEST, the experimental group improved from baseline to post‐test (*p* < 0.001 for both) and remained higher than the control group at post‐test and follow‐up (*p* ≤ 0.001 for all comparisons) (see Tables S2 and S4). For SMS, the experimental group improved from baseline (*p* < 0.001), but between‐group differences at post‐test and follow‐up were not significant (see Table S3). Estimated marginal means with 95% confidence intervals are reported in the Supporting Information. The results of the ANCOVA are presented in Figure 2.

Line graphs display estimated marginal means for WHO‐5, SMS, QUEST 2.0 and SUS at baseline (T0), post‐test (T1) and follow‐up (T2) for the experimental and control groups. In line with the ANCOVA results in Table 3, the experimental group showed larger gains than the control group on WHO‐5 and SUS from T0 to T1, with partial attenuation at T2 but means remaining above the control group. QUEST 2.0 and SMS showed smaller changes than WHO‐5 and SUS. Interactions of time with the covariates (anxiety and stress) were not significant in the ANCOVA models (Table 3), indicating that the observed group differences were not contingent on these variables. Error bars represent 95% confidence intervals.

## 4 Discussion

This study examined the effects of VR‐assisted meditation on psychological well‐being, state mindfulness and technology‐related user experience among veterans with disabilities. The study adds feasibility and implementation‐relevant evidence in a relatively small group that is difficult to recruit for research and provides planning‐level estimates for subsequent trials. The intervention group showed higher psychological well‐being and perceived system usability than the waitlist control. Because the sample was subclinical, outcomes were self‐reported, follow‐up was brief and there was no active comparator, the results should not be interpreted as evidence of clinical efficacy. Generalization to veterans with clinician‐verified PTSD or major depressive disorder is not warranted on the basis of the present design. Although these gains decreased somewhat at follow‐up, they remained higher than those observed in the control group. We also observed improvements in technology satisfaction and mindfulness, but these changes were smaller in magnitude. The overall pattern remained similar after adjusting for baseline anxiety and stress, which reduces although baseline imbalance cannot be ruled out concern about baseline imbalance.

The observed improvement in psychological well‐being suggests a short‐term benefit of VR‐assisted meditation in this sample. One plausible explanation is that positive outcomes were partly linked to the usability of the system and the overall user experience. When participants perceive a system as intuitive and easy to use, they may be more willing to engage with the intervention consistently and with less frustration. This type of engagement may matter because it creates conditions under which users can practice with less friction and accumulate mastery experiences—one of the strongest sources of self‐efficacy in Bandura's framework (Bandura 1997). In this context, a controlled and immersive environment may support willingness to practice and perceived capability. This is a plausible pathway, but it was not directly tested in this study and should be evaluated in mechanism‐focused trials. These findings are consistent with prior VR‐based work in military samples (Botella et al. 2015; Rawlins et al. 2021; Rizzo and Shilling 2017; Van Doren et al. 2024). Beyond usability, the content of the VR experience may also have contributed. Nature‐based virtual scenes may promote affective balance and dampen physiological stress responses (Navarro‐Haro et al. 2017; Seabrook et al. 2020), which could reinforce subjective well‐being during and after practice. At the same time, the decline at follow‐up suggests that maintaining improvements likely depends on continued practice and integration into daily routines. This pattern is consistent with broader behavioural medicine research showing that early gains often attenuate when newly learned skills are not sustained over time (Van Baak and Mariman 2025).

The increase in mindfulness scores in the intervention group suggests that an immersive VR setting can support present‐moment awareness, and that improvements in technology acceptance may occur alongside psychological gains. At the same time, the size and durability of the mindfulness effect were limited, which is consistent with how mindfulness skills typically develop. Mindfulness tends to strengthen gradually through repeated practice, whereas brief programmes often produce more short‐term changes than sustained improvements (Kabat‐Zinn 1990; Navarro‐Haro et al. 2017). Carmody and Baer's (2008) meta‐analysis also shows that outcomes generally improve as programme duration and practice intensity increase. Recent work suggests that VR may not consistently outperform other delivery formats (e.g., tablets) for reducing anxiety. However, VR may offer a different advantage: it can alter the subjective experience of time and promote a stronger sense of flow, which may support adherence over longer periods (Olasz et al. 2024). In line with this idea, the sense of presence in VR may strengthen momentary awareness during sessions, but transferring these gains into daily life likely depends on continued practice after the intervention ends (Navarro‐Haro et al. 2017). This pattern supports the view that mindfulness is a continuity‐dependent skill, and that brief interventions may have limited long‐term effects unless practice is maintained (Gallegos et al. 2017). Regular follow‐up practice may therefore be important not only for sustaining mindfulness gains but also for maintaining improvements in well‐being and positive attitudes toward technology.

From a technology perspective, the observed increases in system usability and satisfaction suggest that the platform was perceived as easy to use, which is consistent with prior research in VR‐based mental health applications (Lindner et al. 2019; Seabrook et al. 2020). The use of the wireless and relatively lightweight Meta Quest 2 headset, along with the customizable Guided Meditation VR software, may have reduced session‐level burden (set‐up time, discomfort and fatigue) and supported repeated use. Its self‐contained set‐up may also have supported ease of use during repeated sessions. In addition, coherent audiovisual environments and reduced exposure to external distractions may have increased engagement and contributed to higher satisfaction (Slater and Sanchez‐Vives 2016). Finally, the combination of usability and satisfaction may have strengthened participants' willingness to engage with the intervention, which may have supported engagement with sessions through improved adherence and a more positive overall experience.

Delivering evidence‐based meditation protocols through an accessible VR platform may produce meaningful short‐term psychological benefits for veterans with disabilities, including those who may benefit from structured, self‐directed practice formats. At the same time, the longer‐term maintenance of these gains is likely to depend on whether early improvements—particularly those supported by positive technology experiences and self‐efficacy—translate into continued practice beyond the structured intervention period. Taken together, the results are best interpreted as preliminary signals that support trial planning, rather than as a basis for best practice recommendations.

## 5 Conclusion

We evaluated the feasibility, acceptability and short‐term outcomes of VR‐assisted meditation among veterans with disabilities. The study was designed as a preliminary trial rather than a test of clinical efficacy.

Overall, the findings suggest that VR‐assisted meditation is a feasible and well‐accepted option in this population. After adjusting for baseline anxiety and stress, the pattern of effects was similar; this does not establish causality but reduces concern that results were driven by baseline distress alone. The immersive nature of VR may also be clinically relevant, as it can reduce external distractions and support sustained attention during practice—an advantage for individuals who find conventional meditation difficult to maintain.

This study provides feasibility data and baseline effect‐size estimates for VR‐assisted meditation in this population. VR may offer a structured practice setting that some participants find easier to engage with. The findings also raise the possibility that VR‐assisted meditation may operate through mechanisms such as increased self‐efficacy and improved willingness to engage with practice in a structured, controllable setting. These findings do not support clinical recommendations; they inform the design of larger trials with active comparators and longer follow‐up.

A brief VR‐assisted meditation programme was associated with improved psychological well‐being, with attenuation at follow‐up. By defining clear feasibility parameters, specific tolerability rates and baseline effect‐size estimates in a relatively small, distinctive group of veterans with disabilities that is difficult to recruit for research, this trial provides planning‐level evidence for subsequent studies. Future trials can use these estimates to justify longer intervention periods, larger sample sizes and the inclusion of physiological indicators to support fully powered VR‐assisted interventions within military rehabilitation contexts. These estimates should be treated as preliminary, given the small sample, waitlist control, reliance on self‐report outcomes and brief follow‐up; they are most useful for trial planning rather than for theory refinement or best‐practice recommendations.

## 6 Implications

The study adds feasibility and implementation‐relevant evidence on VR‐assisted meditation for veterans with disabilities. Rather than positioning the programme as a solution to care access, the study evaluates a structured, self‐guided format that can complement usual care within the practical realities of the US health system. The study suggests that VR can be used to deliver guided mind–body practice, in addition to exposure‐oriented applications. The current study does not introduce new VR hardware or a novel psychological theory. Instead, it evaluates the delivery of an existing guided meditation programme via a consumer VR platform in a sample of veterans with disabilities.

From a theoretical standpoint, the results point to several relevant mechanisms. First, the findings are broadly consistent with Attentional Control Theory and contemporary models of mindfulness: Immersive VR may help users allocate attention toward internal experience while reducing the cognitive demands created by external distractions. Second, the pattern is compatible with a self‐efficacy account, but self‐efficacy was not measured and should be tested directly. Learning meditation in a safe and controllable VR environment may promote mastery experiences, strengthen efficacy beliefs and ultimately support psychological well‐being. Third, the sense of presence in VR may reduce distractibility and support momentary attention during practice. If inhibitory learning processes are relevant, they could be examined in future work using active comparators and preregistered mechanism measures (e.g., expectancy violation indices, behavioural avoidance tasks or psychophysiological markers).

More broadly, the data generate testable hypotheses about how VR may interact with emotion‐regulation processes. Immersive environments may reduce the cognitive load associated with early mindfulness practice, making the learning process feel more manageable and making early practice feel more manageable for skill acquisition. The findings also support an embodied perspective: VR's sensory richness may help turn an abstract instruction (‘notice your breath’) into a more concrete and accessible experience. This possibility should be tested in clinical samples using clinician‐verified assessments. Under this view, the potential benefits may reflect not only attentional control but also a gradual process of re‐embodiment.

From a practical perspective, VR‐assisted meditation may be considered as a structured, self‐guided format that could complement usual care; comparative trials are needed before making clinical recommendations. The format may also reduce stigma and support engagement, especially among veterans who are comfortable with digital tools and self‐directed learning. For developers, the findings highlight an important point: Usability alone is not sufficient. Therapeutic VR applications should combine usable design with evidence‐based content and evaluation.

At a societal and policy level, the results may warrant small‐scale implementation pilots with outcome monitoring, rather than broad procurement or system‐wide rollout. Any implementation beyond research settings would require staged pilots with monitoring and evidence from longer trials with active comparators. At the same time, evidence for long‐term effectiveness is still limited. If implementation pilots are considered, they should include outcome monitoring and clear guidance on privacy and safe use.

Taken together, the study suggests a feasible delivery option with short‐term benefits that merits replication in larger, longer trials with active comparators and mechanism measures. The main contributions are feasibility evidence and planning‐level estimates that can inform fully powered trials. Further trials are needed before VR‐based mindfulness can be recommended as part of routine care.

## 7 Limitations and Directions for Future Research

Several limitations should be considered when interpreting these results. First, the study used a relatively small and fairly homogeneous sample, which reduces statistical power and limits the extent to which the results can be applied to veterans with different demographic or clinical characteristics. Second, the design did not include an active comparator condition. As a result, it is difficult to determine whether the observed effects were primarily driven by VR‐specific features (e.g., immersion and presence) or by meditation practice itself. Third, outcomes relied mainly on self‐report measures, which are vulnerable to response biases such as social desirability and may not fully reflect underlying physiological change. Fourth, follow‐up was limited, which prevents stronger conclusions about whether benefits persist beyond the short term. Finally, purposive and snowball recruitment strategies may have increased self‐selection effects and reduced sample representativeness.

These limitations point to several directions for future research. Larger, adequately powered randomized trials with more heterogeneous samples are needed, including veterans from different service eras and with varying disability types and comorbidity profiles. Future studies should also incorporate active control groups—such as conventional guided meditation, tablet‐based mindfulness programmes, or other evidence‐based interventions—to better isolate the specific added value of VR. Mixed‐methods designs that integrate standardized outcome measures with qualitative interviews could provide richer insight into user experience, acceptability and implementation barriers. In addition to self‐report, incorporating physiological markers (e.g., heart‐rate variability and salivary cortisol) would allow more objective assessment of stress regulation and recovery processes. Finally, testing mediators and moderators—such as self‐efficacy, sense of presence, individual difference factors, social support and baseline attitudes toward technology—would help clarify for whom VR‐assisted meditation works best and through which mechanisms. These steps would clarify durability, comparative benefit and mechanisms.

## Funding

The author U.D. was supported by the Scientific and Technological Research Council of Türkiye (TÜBİTAK) under the 2219—International Postdoctoral Research Fellowship Program (Application No: 1059B192301468). The content is solely the responsibility of the authors and does not necessarily represent the official views of TÜBİTAK.

## Ethics Statement

The study protocol received approval from the Michigan State University Social/Behavioral/Education Institutional Review Board (IRB) on 27 November 2024 (Protocol No: STUDY00011212). Written informed consent was obtained from all participants prior to enrolment. The study adhered to the principles of the Declaration of Helsinki.

## Conflicts of Interest

The authors declare no conflicts of interest.

## Supporting information




**Table S1:** Post hoc comparisons for World Health Organization–Five Well‐Being Index (WHO‐5) scores.
**Table S2:** Post hoc comparisons for Quebec User Evaluation of Satisfaction with Assistive Technology (QUEST 2.0) scores.
**Table S3:** Post hoc comparisons for State Mindfulness Scale (SMS) scores.
**Table S4:** Post hoc comparisons for System Usability Scale (SUS) scores.

## Data Availability

The data that support the findings of this study are available from the corresponding author (U.D.) upon reasonable request. The data are not publicly available due to privacy and ethical restrictions regarding the participants.
