# Supplementary material for: Mindfulness in Technology: Feasibility and Preliminary Efficacy of VR‐Assisted Meditation Among Veterans With Disabilities
Source: Clin Psychol Psychother. 2026 Apr 4;33(2):e70266. doi: 10.1002/cpp.70266 (PMC13050182; doi:10.1002/cpp.70266)
Supplement: Supplementary file 1 — Table S1: Post hoc comparisons for World Health Organization–Five Well‐Being Index (WHO‐5) scores. Table S2: Post hoc comparisons for Quebec User Evaluation of Satisfaction with Assistive Technology (QUEST 2.0) scores. Table S3: Post hoc comparisons for State Mindfulness Scale (SMS) scores. Table S4: Post hoc comparisons for System Usability Scale (SUS) scores. [file CPP-33-e70266-s001.docx]

| Table S1. Post Hoc Comparisons for World Health Organization-Five Well-Being Index (WHO-5) Scores | | | | | | | | | |  |  |
| --- | --- | --- | --- | --- | --- | --- | --- | --- | --- | --- | --- |
| **Measures** | **Group** |  | **Measures** | **Group** | **Mean Difference** | **SE** | **df** | **t** | **p_tukey_** | **95% CI Lower** | **95% CI Upper** |
| Baseline | Experimental | - | Baseline | Control | -0,41 | 0,57 | 38 | -0,72 | 0,978 | -1,56 | 0,74 |
|  |  | - | Post-test | Experimental | -1,42 | 0,32 | 38 | -4,46 | < ,001 | -2,07 | -0,77 |
|  |  | - | Post-test | Control | -0,08 | 0,5 | 38 | -0,15 | 1 | -1,09 | 0,93 |
|  |  | - | Follow-up | Experimental | -0,79 | 0,32 | 38 | -2,48 | 0,155 | -1,44 | -0,14 |
|  |  | - | Follow-up | Control | -0,17 | 0,53 | 38 | -0,32 | 0,999 | -1,24 | 0,90 |
|  | Control | - | Post-test | Experimental | -1,01 | 0,5 | 38 | -2,04 | 0,342 | -2,02 | 0,00 |
|  |  | - | Post-test | Control | 0,33 | 0,3 | 38 | 1,1 | 0,878 | -0,28 | 0,94 |
|  |  | - | Follow-up | Experimental | -0,38 | 0,53 | 38 | -0,72 | 0,979 | -1,45 | 0,69 |
|  |  | - | Follow-up | Control | 0,24 | 0,3 | 38 | 0,79 | 0,968 | -0,37 | 0,85 |
| Post-test | Experimental | - | Post-test | Control | 1,34 | 0,42 | 38 | 3,23 | 0,029 | 0,49 | 2,19 |
|  |  | - | Follow-up | Experimental | 0,63 | 0,3 | 38 | 2,09 | 0,316 | 0,02 | 1,24 |
|  |  | - | Follow-up | Control | 1,25 | 0,45 | 38 | 2,77 | 0,084 | 0,34 | 2,16 |
|  | Control | - | Follow-up | Experimental | -0,71 | 0,45 | 38 | -1,57 | 0,623 | -1,62 | 0,20 |
|  |  | - | Follow-up | Control | -0,1 | 0,29 | 38 | -0,33 | 0,999 | -0,69 | 0,49 |
| Follow-up | Experimental | - | Follow-up | Control | 0,62 | 0,49 | 38 | 1,27 | 0,799 | -0,37 | 1,61 |

| Estimated Marginal Means - Measures ✻ Group | | | | | |
| --- | --- | --- | --- | --- | --- |
|  | | | | **95% CI** | |
| **Group** | **Measures** | **Mean** | **SE** | **Lower** | **Upper** |
| EXPRE | Pretest | 38,33 | 1,58 | 35,12 | 41,54 |
|  | Posttest | 44,31 | 1,19 | 41,88 | 46,73 |
|  | Follow-up | 41,51 | 1,29 | 38,90 | 44,13 |
| CONTROL | Pretest | 39,80 | 1,50 | 36,75 | 42,85 |
|  | Posttest | 39,15 | 1,14 | 36,85 | 41,45 |
|  | Follow-up | 39,77 | 1,23 | 37,29 | 42,26 |

| Table S2. Post Hoc Comparisons for Quebec User Evaluation of Satisfaction with Assistive Technology (QUEST 2,0) Scores | | | | | | | | | |  |  |
| --- | --- | --- | --- | --- | --- | --- | --- | --- | --- | --- | --- |
| **Measures** | **Group** |  | **Measures** | **Group** | **Mean Difference** | **SE** | **df** | **t** | **p_tukey_** | **95% CI Lower** | **95% CI Upper** |
| Baseline | Experimental | - | Baseline | Control | 0,31 | 0,49 | 36 | 0,63 | 0,988 | -0,68 | 1,30 |
|  |  | - | Posttest | Experimental | -2,71 | 0,25 | 36 | -10,67 | < ,001 | -3,22 | -2,21 |
|  |  | - | Posttest | Control | 0,14 | 0,47 | 36 | 0,30 | 1,000 | -0,81 | 1,09 |
|  |  | - | Follow-up | Experimental | -1,6 | 0,34 | 36 | -4,65 | < ,001 | -2,29 | -0,91 |
|  |  | - | Follow-up | Control | 0,75 | 0,49 | 36 | 1,54 | 0,643 | -0,24 | 1,74 |
|  | Control | - | Posttest | Experimental | -3,02 | 0,47 | 36 | -6,48 | < ,001 | -3,97 | -2,07 |
|  |  | - | Posttest | Control | -0,17 | 0,24 | 36 | -0,69 | 0,982 | -0,65 | 0,31 |
|  |  | - | Follow-up | Experimental | -1,9 | 0,49 | 36 | -3,89 | 0,005 | -2,89 | -0,91 |
|  |  | - | Follow-up | Control | 0,44 | 0,33 | 36 | 1,36 | 0,75 | -0,23 | 1,11 |
| Posttest | Experimental | - | Posttest | Control | 2,85 | 0,45 | 36 | 6,37 | < ,001 | 1,94 | 3,76 |
|  |  | - | Follow-up | Experimental | 1,11 | 0,24 | 36 | 4,72 | < ,001 | 0,63 | 1,59 |
|  |  | - | Follow-up | Control | 3,46 | 0,47 | 36 | 7,36 | < ,001 | 2,51 | 4,41 |
|  | Control | - | Follow-up | Experimental | -1,74 | 0,47 | 36 | -3,68 | 0,009 | -2,69 | -0,79 |
|  |  | - | Follow-up | Control | 0,61 | 0,22 | 36 | 2,73 | 0,095 | 0,17 | 1,05 |
| Follow-up | Experimental | - | Follow-up | Control | 2,35 | 0,5 | 36 | 4,74 | < ,001 | 1,34 | 3,36 |

| Estimated Marginal Means - Measures ✻ Group | | | | | |
| --- | --- | --- | --- | --- | --- |
|  | | | | **95% CI** | |
| **Grup** | **Measures** | **Mean** | **SE** | **Lower** | **Upper** |
| EXPRE | Pretest | 55,46 | 0,35 | 54,75 | 56,17 |
|  | Posttest | 58,17 | 0,32 | 57,52 | 58,83 |
|  | Follow-up | 57,06 | 0,36 | 56,33 | 57,78 |
| CONTROL | Pretest | 55,15 | 0,33 | 54,48 | 55,83 |
|  | Posttest | 55,32 | 0,31 | 54,70 | 55,94 |
|  | Follow-up | 54,71 | 0,34 | 54,02 | 55,40 |

|  |
| --- |

| Table S3. Post Hoc Comparisons for State Mindfulness Scale (SMS) Scores | | | | | | | | | |  |  |
| --- | --- | --- | --- | --- | --- | --- | --- | --- | --- | --- | --- |
| **Measures** | **Group** |  | **Measures** | **Group** | **Mean Difference** | **SE** | **df** | **t** | **p_tukey_** | **95% CI Lower** | **95% CI Upper** |
| Baseline | Experimental | - | Baseline | Control | -1,84 | 1,54 | 36 | -1,19 | 0,838 | -4,95 | 1,27 |
|  |  | - | Posttest | Experimental | -3,05 | 0,65 | 36 | -4,69 | < ,001 | -4,36 | -1,74 |
|  |  | - | Posttest | Control | -1,98 | 1,67 | 36 | -1,19 | 0,840 | -5,35 | 1,39 |
|  |  | - | Follow-up | Experimental | -2,71 | 0,36 | 36 | -7,45 | < ,001 | -3,44 | -1,98 |
|  |  | - | Follow-up | Control | -4,19 | 1,57 | 36 | -2,67 | 0,108 | -7,36 | -1,02 |
|  | Control | - | Posttest | Experimental | -1,21 | 1,68 | 36 | -0,72 | 0,978 | -4,60 | 2,18 |
|  |  | - | Posttest | Control | -0,14 | 0,62 | 36 | -0,23 | 1,000 | -1,39 | 1,11 |
|  |  | - | Follow-up | Experimental | -0,88 | 1,58 | 36 | -0,56 | 0,993 | -4,07 | 2,31 |
|  |  | - | Follow-up | Control | -2,35 | 0,35 | 36 | -6,80 | < ,001 | -3,06 | -1,64 |
| Posttest | Experimental | - | Posttest | Control | 1,07 | 1,8 | 36 | 0,60 | 0,991 | -2,57 | 4,71 |
|  |  | - | Follow-up | Experimental | 0,34 | 0,68 | 36 | 0,50 | 0,996 | -1,03 | 1,71 |
|  |  | - | Follow-up | Control | -1,14 | 1,71 | 36 | -0,67 | 0,984 | -4,59 | 2,31 |
|  | Control | - | Follow-up | Experimental | -0,73 | 1,7 | 36 | -0,43 | 0,998 | -4,16 | 2,70 |
|  |  | - | Follow-up | Control | -2,21 | 0,65 | 36 | -3,40 | 0,019 | -3,52 | -0,90 |
| Follow-up | Experimental | - | Follow-up | Control | -1,48 | 1,61 | 36 | -0,92 | 0,938 | -4,73 | 1,77 |

| Estimated Marginal Means - Measures ✻ Group | | | | | |
| --- | --- | --- | --- | --- | --- |
|  | | | | **95% CI** | |
| **Group** | **Measures** | **Mean** | **SE** | **Lower** | **Upper** |
| EXPRE | Pretest | 37,69 | 1,11 | 35,43 | 39,94 |
|  | Posttest | 40,74 | 1,30 | 38,10 | 43,37 |
|  | Follow-up | 40,40 | 1,16 | 38,05 | 42,75 |
| CONTROL | Pretest | 39,52 | 1,06 | 37,38 | 41,67 |
|  | Posttest | 39,67 | 1,23 | 37,16 | 42,17 |
|  | Follow-up | 41,88 | 1,10 | 39,64 | 44,11 |

| Table S4. Post Hoc Comparisons for System Usability Scale (SUS) Scores | | | | | | | | | |  |  |
| --- | --- | --- | --- | --- | --- | --- | --- | --- | --- | --- | --- |
| **Measures** | **Group** |  | **Measures** | **Group** | **Mean Difference** | **SE** | **df** | **t** | **p_tukey_** | **95% CI Lower** | **95% CI Upper** |
| Baseline | Experimental | - | Baseline | Control | 0,48 | 2,6 | 36 | 0,18 | 1,000 | -4,79 | 5,75 |
|  |  | - | Posttest | Experimental | -12,91 | 1,1 | 36 | -11,7 | < ,001 | -15,15 | -10,67 |
|  |  | - | Posttest | Control | 0,75 | 2,6 | 36 | 0,29 | 1,000 | -4,54 | 6,04 |
|  |  | - | Follow-up | Experimental | -10,81 | 1,8 | 36 | -5,92 | < ,001 | -14,51 | -7,11 |
|  |  | - | Follow-up | Control | 2,16 | 2,8 | 36 | 0,77 | 0,970 | -3,48 | 7,80 |
|  | Control | - | Posttest | Experimental | -13,39 | 2,6 | 36 | -5,11 | < ,001 | -18,68 | -8,10 |
|  |  | - | Posttest | Control | 0,27 | 1,1 | 36 | 0,26 | 1,000 | -1,85 | 2,39 |
|  |  | - | Follow-up | Experimental | -11,29 | 2,8 | 36 | -4,01 | 0,004 | -16,97 | -5,61 |
|  |  | - | Follow-up | Control | 1,68 | 1,7 | 36 | 0,97 | 0,924 | -1,83 | 5,19 |
| Posttest | Experimental | - | Posttest | Control | 13,66 | 2,6 | 36 | 5,19 | < ,001 | 8,35 | 18,97 |
|  |  | - | Follow-up | Experimental | 2,10 | 1,6 | 36 | 1,28 | 0,792 | -1,21 | 5,41 |
|  |  | - | Follow-up | Control | 15,07 | ### | 36 | 5,37 | < ,001 | 9,41 | 20,73 |
|  | Control | - | Follow-up | Experimental | -11,56 | 2,8 | 36 | -4,10 | 0,003 | -17,26 | -5,86 |
|  |  | - | Follow-up | Control | 1,41 | 1,6 | 36 | 0,91 | 0,942 | -1,74 | 4,56 |
| Follow-up | Experimental | - | Follow-up | Control | 12,97 | 3 | 36 | 4,34 | 0,001 | 6,93 | 19,01 |

| Estimated Marginal Means - Measures ✻ Group | | | | | |
| --- | --- | --- | --- | --- | --- |
|  | | | | **95% CI** | |
| **Group** | **Measures** | **Mean** | **SE** | **Lower** | **Upper** |
| EXPRE | Pretest | 73,06 | 1,89 | 69,24 | 76,89 |
|  | Posttest | 85,97 | 1,90 | 82,12 | 89,82 |
|  | Follow-up | 83,87 | 2,16 | 79,49 | 88,25 |
| CONTROL | Pretest | 72,59 | 1,79 | 68,95 | 76,22 |
|  | Posttest | 72,31 | 1,81 | 68,65 | 75,97 |
|  | Follow-up | 70,90 | 2,05 | 66,74 | 75,06 |
